# Inhibition of Cdk5 in PV Neurons Reactivates Experience-Dependent Plasticity in Adult Visual Cortex

**DOI:** 10.3390/ijms23010186

**Published:** 2021-12-24

**Authors:** Xinxin Zhang, Huiping Tang, Sitong Li, Yueqin Liu, Wei Wu, Yue Li, Chenchen Ma, Xiao Ma, Lin Chen, Yupeng Yang

**Affiliations:** Hefei National Laboratory for Physical Sciences at the Microscale, CAS Key Laboratory of Brain Function and Disease, Division of Life Sciences and Medicine, School of Life Sciences, University of Science and Technology of China, Hefei 230027, China; zxx4568@mail.ustc.edu.cn (X.Z.); thp0425@mail.ustc.edu.cn (H.T.); sitongli1993@gmail.com (S.L.); yql918@mail.ustc.edu.cn (Y.L.); wuw@mail.ustc.edu.cn (W.W.); liyue7510@gmail.com (Y.L.); machen96@mail.ustc.edu.cn (C.M.); moheart666@outlook.com (X.M.); linchen@ustc.edu.cn (L.C.)

**Keywords:** Cdk5, PV neurons, ocular dominance plasticity, primary visual cortex, monocular deprivation

## Abstract

Cyclin-dependent kinase 5 (Cdk5) has been shown to play a critical role in brain development, learning, memory and neural processing in general. Cdk5 is widely distributed in many neuron types in the central nervous system, while its cell-specific role is largely unknown. Our previous study showed that Cdk5 inhibition restored ocular dominance (OD) plasticity in adulthood. In this study, we specifically knocked down Cdk5 in different types of neurons in the visual cortex and examined OD plasticity by optical imaging of intrinsic signals. Downregulation of Cdk5 in parvalbumin-expressing (PV) inhibitory neurons, but not other neurons, reactivated adult mouse visual cortical plasticity. Cdk5 knockdown in PV neurons reduced the evoked firing rate, which was accompanied by an increment in the threshold current for the generation of a single action potential (AP) and hyperpolarization of the resting membrane potential. Moreover, chemogenetic activation of PV neurons in the visual cortex can attenuate the restoration of OD plasticity by Cdk5 inhibition. Taken together, our results suggest that Cdk5 in PV interneurons may play a role in modulating the excitation and inhibition balance to control the plasticity of the visual cortex.

## 1. Introduction

Cyclin-dependent kinase 5 (Cdk5) is a proline-directed serine/threonine kinase expressed abundantly in post-mitotic neurons and plays a vital role in different brain regions [1,2]. It has been reported that overactivity of Cdk5 is associated with neurodegenerative diseases such as Alzheimer’s disease (AD), Parkinson’s disease and Huntington’s disease [2,3,4]. Hyperactive Cdk5 in the striatum results in loss of cocaine sensitization and motor coordination deficits [5]. Meanwhile, pharmacological inhibition of Cdk5 in the nucleus accumbens enhances the locomotor-activating and incentive-motivational effects of cocaine [6]. RNA interference of Cdk5 in the hippocampus prevents cerebral ischemia-induced neurodegeneration and cognitive dysfunction [7]. Similar treatment in hippocampal CA1 increases long-term potentiation (LTP) in young mice and recovers paired-pulse facilitation and LTP in a mouse model of AD [8]. The role of Cdk5 in the visual cortex is largely unknown. Downregulation of Cdk5 activity by pharmacological inhibition or genetic knockdown rejuvenates the visual cortex and enhances the synaptic plasticity of the adult mouse visual cortex [9], but the underlying mechanism remains unclear.

Cdk5 is widely distributed in various cells in the brain and exerts its function in a cell-specific manner. After ablation of Cdk5 in excitatory pyramidal neurons of the forebrain, mice exhibit mania-like behavior and impaired performance in fear conditioning and Morris water maze tasks [10]. Selective ablation of Cdk5 in PV interneurons from birth leads to an increase in gamma-aminobutyric acid (GABA) neurotransmission in CA1 pyramidal neurons and cognitive impairment in young adult mice [11]. Furthermore, silencing Cdk5 in astrocytes exerts neuroprotective effects through promoting the activation of a small Rho guanosine triphosphatases, Rac1, and cell stellation [12]. The specific deletion of Cdk5 in oligodendrocytes disrupts the architecture of nodes of Ranvier and causes impaired differentiation of oligodendrocytes [13,14]. However, the functional differences of Cdk5 among different cell types in the same brain region have not been systematically studied.

In the early stages of development, the neural circuits are rapidly rewired and refined in response to the changing stimuli of the external environment. Monocular deprivation (MD) of pattern vision triggers a robust reduction in the responses of binocular visual cortical neurons to the deprived eye, which is termed ocular dominance (OD) plasticity. As the brain matures, the adaptation of brain functions, including visual function, becomes weaker [15,16]. Most treatments for adult amblyopia focus on restoring visual plasticity [17,18]. Both excitatory and inhibitory circuits play an important role in the rejuvenation of the visual cortex in adults. The reactivation of OD plasticity is often accompanied by a significant increase in the proportion of the GluN2B subunit of the N-methyl-D-aspartate (NMDA) receptor [19,20]. Moreover, a direct or indirect reduction in GABAergic inhibition in adults promotes the restoration of OD plasticity [17,18,21,22,23,24]. We have shown that non-specific knockdown of Cdk5 in the visual cortex induces a significant increase in GluN2B-mediated currents in excitatory neurons and a reduction in GABAergic input from inhibitory neurons [9]. It is worthwhile to identify cell types through which Cdk5 exerts its regulatory functions on visual plasticity.

In the visual cortex, three main non-overlapping classes of cortical interneurons: those expressing parvalbumin (PV), somatostatin (SST) and vasoactive intestinal peptide (VIP), together with pyramidal neurons, are involved in visual plasticity [25,26]. In this study, we specifically knocked down Cdk5 in neuronal subtypes by injecting AAV–LSL–shCdk5 or AAV–LSL–scramble, a Cre-inducible AAV, into the primary visual cortex (V1) of different Cre transgenic mice. Using optical imaging of intrinsic signals in V1, we found that downregulation of Cdk5 activity in PV neurons, but not other cell types, facilitated a remarkable restoration of OD plasticity in adults, which was mediated by the downregulation of the inhibitory activity of PV neurons. These results suggest that the PV-based inhibitory circuit is the main target for Cdk5 to regulate the visual plasticity of adult mice.

## 2. Results

### 2.1. Cdk5 Is Co-Labeled with a Variety of Neurons in the Visual Cortex, and Pharmacological Inhibition of Cdk5 Restores OD Plasticity in Adult WT Mice

**Figure 1:**
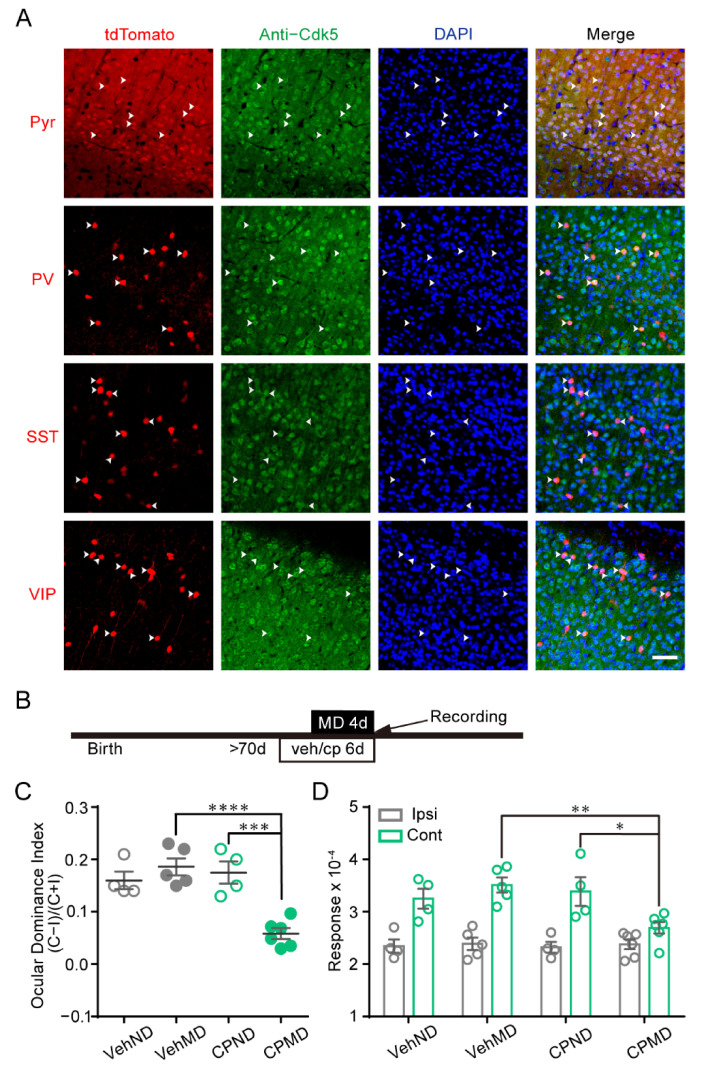
The distribution of Cdk5 in subtype neurons of mouse V1, and the reactivation of OD plasticity by pharmacological inhibition of Cdk5. (**A**) Representative images of the co-localization of tdTomato-expressing neurons (pyramidal (Pyr), parvalbumin (PV), somatostatin (SST) or vasoactive intestinal polypeptide (VIP) neurons) (red) with Cdk5-positive cells (green) in V1. Arrows indicate the co-localized neurons of tdTomato expression and molecular markers. Scale bar, 50 μm. (**B**) Experimental design. (**C**) Comparison of ODI measured from vehicle-treated mice with/without MD (gray) and CP681301-treated mice with/without MD (green) (F(1, 15) = 12.66; CPMD versus CPND, *p* < 0.001; CPMD versus VehMD, *p* < 0.0001; two-way ANOVA with Tukey’s post hoc test). (**D**) Comparison of response magnitude of intrinsic optical signals elicited by stimulating the ipsilateral eye or contralateral eye (F(3, 30) = 3.51; cont: CPMD versus CPND, *p* < 0.05; CPMD versus VehMD, *p* < 0.01; two-way ANOVA with Tukey’s post hoc test). Veh: vehicle; CP: CP681301; ODI: ocular dominance index; MD: monocular deprivation; ipsi: ipsilateral eye; cont: contralateral eye. Data shown as mean ± SEM. * *p* < 0.05, ** *p* < 0.01, *** *p* < 0.001, **** *p* < 0.0001.

Cdk5 is widely distributed in various tissues, but its kinase activity is restricted in neurons [27,28]. To identify the expression of Cdk5 in neuronal subtypes in the visual cortex, we generated CaMKII, PV, SST or VIP–IRES–Cre; Ai14 mice, which specifically expressed tdTomato in pyramidal (Pyr), PV-positive, SST-positive or VIP-positive neurons, respectively. Cdk5 was labeled green by immunofluorescence. We found that most of the tdTomato expression neurons co-labeled with Cdk5 in the visual cortex (Figure 1A). This result suggests that Cdk5 is highly expressed in general neuronal subtypes of the visual cortex.

Our previous study using single-unit recording showed that pharmacological administration of a Cdk5 inhibitor can evoke OD plasticity in the adult mouse visual cortex [9]. This effect of Cdk5 on the OD plasticity of adult WT mice was further investigated using intrinsic signal optical imaging. OD plasticity of V1 was measured in mice receiving vehicle or CP681301, which is a specific, brain-permeable inhibitor of Cdk5 [29] (Figure 1B). The imaging of intrinsic signals was produced by presentation of visual stimuli in the binocular visual field (−5°–15° azimuth) to each eye (Supplementary Figure S1). The ocular dominance index (ODI) was calculated by averaging the ratios of intrinsic signals evoked by visual stimuli to the ipsilateral eye (ipsi) and the contralateral eye (cont) [16,30]. After 4d MD, the mean ODI of mice injected with CP681301 was significantly lower than that of vehicle-treated mice (VehND = 0.16 ± 0.02, n = 4; VehMD = 0.19 ± 0.02, n = 5; CPND = 0.18 ± 0.02, n = 4; CPMD = 0.06 ± 0.01, n = 6; two-way ANOVA, *p* < 0.001; Figure 1C). The reduction in the ODI of CP681301-treated mice was mediated by a decrease in the deprived/contralateral eye responses. The non-deprived/ipsilateral eye responses were comparable (cont: VehND = 3.25 ± 0.19; VehMD = 3.51 ± 0.14; CPND = 3.39 ± 0.27; CPMD = 2.70 ± 0.11; ipsi: VehND = 2.35 ± 0.13; VehMD = 2.39 ± 0.12; CPND = 2.32 ± 0.10; CPMD = 2.38 ± 0.10; Figure 1D). The situation was similar to OD plasticity induced by short-term MD during the critical period [16]. These results indicate that inhibition of Cdk5 reinstates juvenile-like OD plasticity in adult mice, which is closely consistent with our previous study [9].

### 2.2. Knockdown of Cdk5 in V1 Pyramidal Neurons Fails to Restore OD Plasticity in Adults

**Figure 2:**
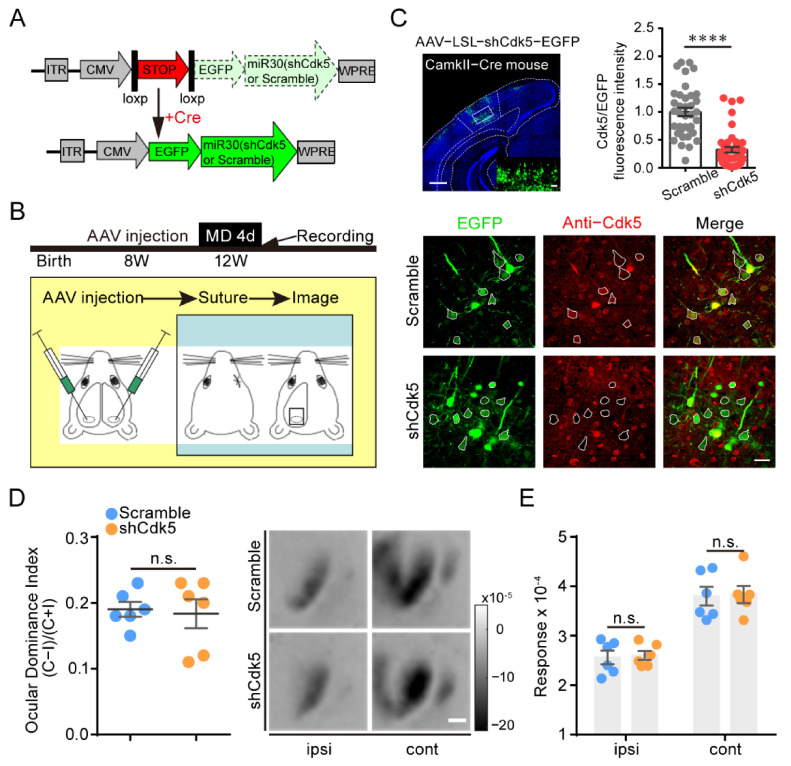
Knockdown of Cdk5 in V1 pyramidal neurons has no effect on adult OD plasticity. (**A**) Scheme for the modification of a miR30 vector (shCdk5 or scramble gene sequence) into an LSL/Cre-controlled version. The conditional miR30 vector can be activated by excision of the stop cassette through Cre-mediated deletion. (**B**) Experimental design. V1L: AAV–shCdk5/scramble; V1R: AAV–scramble. (**C**) Upper left: A representative image of infected pyramidal neurons with AAV–shCdk5. Scale bar, 500 μm. Enlarged view shown in inset. Scale bar, 50 μm. Upper right: Cdk5 was quantified by immunofluorescence using Abs against Cdk5 (scramble = 1.00 ± 0.07, n = 37; shCdk5 = 0.32 ± 0.05, n = 42; t = 7.71, *p* < 0.0001, unpaired *t*-test). Bottom: IHC in V1 of CaMKII-Cre mice. Virus-infected pyramidal neurons (green) and Cdk5 immunoreactivity (red). Virus-infected pyramidal neurons are enclosed by white hollow circles. Scale bar, 30 μm. (**D**) Left: Comparison of ODI measured from CaMKII-Cre mice injected with AAV–scramble (blue) and AAV–shCdk5 (orange) after 4d MD (t = 0.27, *p* = 0.79, unpaired *t*-test). Right: Example intrinsic signal images from CaMKII-Cre mice injected with AAV–scramble and AAV–shCdk5 after MD. Scale bar, 500 μm. (**E**) Comparison of response magnitude of intrinsic optical signals elicited by stimulating the ipsilateral eye or contralateral eye for AAV–scramble- or AAV–shCdk5-injected CaMKII-Cre mice following 4d MD (F(1, 20) = 0.06, *p* > 0.05, two-way ANOVA with Tukey’s post hoc test). ODI: ocular dominance index; MD: monocular deprivation; ipsi: ipsilateral eye; cont: contralateral eye. Data shown as mean ± SEM. n.s., not statistically significant, **** *p* < 0.0001.

Cdk5 inhibition increases the total GluN2B protein level in CA1 [31] and induces an increase in GluN2B-mediated currents in pyramidal neurons in the visual cortex [9]. To determine whether Cdk5 in pyramidal neurons contributes to the restoration of OD plasticity, we injected AAV–LSL–shCdk5, a Cre-inducible AAV expressing a Cdk5miR, into V1 of CaMKII-Cre mice at 8 weeks of age. Four weeks later, OD plasticity was examined after the mice were subjected to four days of MD (Figure 2A,B). The expression of Cdk5 in pyramidal neurons was significantly reduced in mice receiving AAV–LSL–shCdk5 compared with the control scramble virus (shCdk5 = 0.32 ± 0.05; unpaired *t*-test, *p* < 0.0001; Figure 2C). Four days of MD did not induce significant OD plasticity in adult CaMKII-Cre mice with or without Cdk5 downregulation, with a similar ODI in both MD groups (scramble = 0.19 ± 0.01, n = 6; shCdk5 = 0.18 ± 0.02, n = 6; unpaired *t*-test, *p* = 0.79; Figure 2D). Compared with those in AAV-scramble mice, the response amplitudes driven by either the deprived or the non-deprived eyes were not significantly different in AAV-shCdk5 mice (cont: scramble = 3.80 ± 0.19; shCdk5 = 3.83 ± 0.17; ipsi: scramble = 2.56 ± 0.14; shCdk5 = 2.60 ± 0.09; two-way ANOVA, *p* > 0.05; Figure 2E). These findings suggest that knockdown of Cdk5 in V1 pyramidal neurons has no effect on the restoration of OD plasticity in adults.

### 2.3. Knockdown of Cdk5 in PV, but Not SST or VIP Neurons, of V1 Reactivates OD Plasticity in Adult Mice

**Figure 3:**
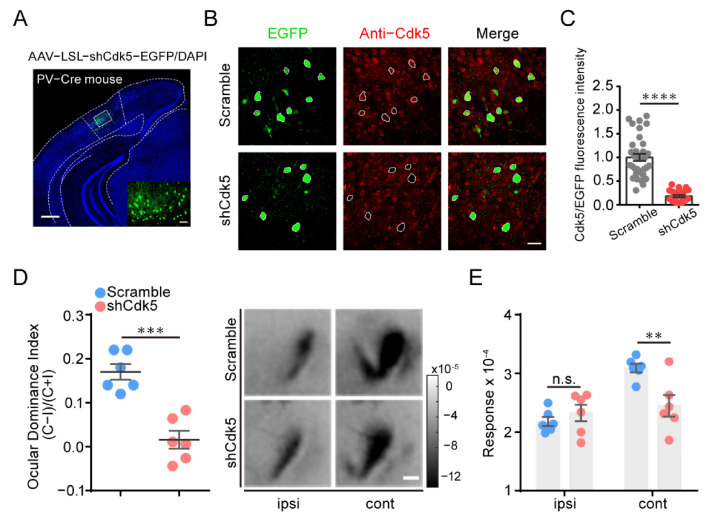
Cdk5 knockdown in PV neurons of V1 is sufficient to reactivate OD plasticity in adult mice. (**A**) A representative image of infected PV neurons with AAV–shCdk5. Scale bar, 500 μm. Enlarged view shown in inset. Scale bar, 50 μm. (**B**) IHC in V1 of PV-Cre mice, virus-infected PV neurons (green) and Cdk5 immunoreactivity (red). Virus-infected PV neurons are enclosed by white hollow circles. Scale bar, 30 μm. (**C**) Cdk5 was quantified by immunofluorescence using Abs against Cdk5 (scramble = 1.00 ± 0.07, n = 34; shCdk5 = 0.18 ± 0.02, n = 24; t = 8.96, *p* < 0.0001, unpaired *t*-test). (**D**) Left: Optically imaged ODIs in PV-Cre mice with AAV–scramble (blue) or AAV–shCdk5 (erythrinus) expression in V1 (t = 5.74, *p* = 0.0002, unpaired *t*-test). Right: Example intrinsic optical signals seen in V1 for each eye stimulation after MD. Scale bar, 500 μm. (**E**) After 4d MD, V1 activation elicited by stimulation of the ipsilateral eye or contralateral eye from PV-Cre mice infected with AAV–scramble or AAV–shCdk5 (F(1, 20) = 3.84, *p* < 0.01, two-way ANOVA with Tukey’s post hoc test). ODI: ocular dominance index; MD: monocular deprivation; ipsi: ipsilateral eye; cont: contralateral eye. Data shown as mean ± SEM. n.s., not statistically significant, ** *p* < 0.01, *** *p* < 0.001, **** *p* < 0.0001.

Since Cdk5 inhibition rejuvenated inhibitory neurotransmission in adulthood [9], we speculated that the reactivation of OD plasticity in adults may be mediated by Cdk5 downregulation in inhibitory neurons. To test this hypothesis, we first knocked down Cdk5 in PV neurons by injecting AAV–LSL–shCdk5 into the visual cortex of adult PV-Cre mice (Figure 3A). Compared with control scramble mice, the co-expression of Cdk5 and PV interneurons in AAV-shCdk5 mice was reduced to 18% ± 2% (unpaired *t*-test, *p* < 0.0001; Figure 3B,C). Surprisingly, adult mice with Cdk5 knockdown in PV neurons showed OD plasticity induced by four days of MD, with a significantly lower ODI than that of control scramble mice (scramble = 0.17 ± 0.02, n = 6; shCdk5 = 0.02 ± 0.02, n = 6; unpaired *t*-test, *p* < 0.001; Figure 3D). This plasticity was mainly the result of a dramatic decrease in the deprived-eye response (cont: scramble = 3.09 ± 0.07; shCdk5 = 2.45 ± 0.18; ipsi: scramble = 2.18 ± 0.08; shCdk5 = 2.33 ± 0.14; two-way ANOVA, *p* < 0.01; Figure 3E). These results indicate that targeted knockdown of Cdk5 in PV neurons of V1 is sufficient for the reinstatement of visual plasticity in adult mice.

**Figure 4:**
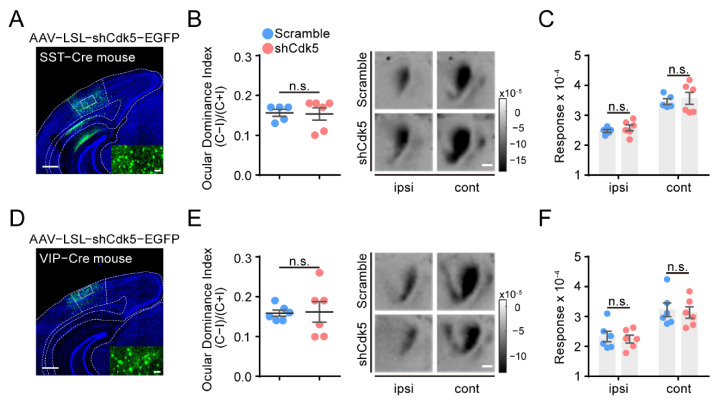
Cdk5 knockdown in SST or VIP neurons of V1 cannot reactivate OD plasticity in adult mice. (**A**,**D**) Representative images of injection with AAV–shCdk5 in the V1 of SST-Cre (**A**) and VIP-Cre (**D**) mice. Scale bar, 500 μm. Enlarged view shown in inset. Scale bar, 50 μm. (**B**) Left: After 4d MD, average ODI measured in adult SST-Cre mice transfected with AAV–scramble (blue) or AAV–shCdk5 (erythrinus) (t = 0.14, *p* = 0.89, unpaired *t*-test). Right: Example intrinsic signal images from SST-Cre mice injected with AAV–scramble and AAV–shCdk5 after MD. Scale bar, 500 μm. (**C**) Maximum magnitude of responses elicited by the stimulus to the ipsilateral eye or contralateral eye after 4d MD in adult SST-Cre mice (F(1, 18) = 0.61, *p* > 0.05, two-way ANOVA with Tukey’s post hoc test). (**E**,**F**) Quantification of visual cortical activation in VIP-Cre mice. ODI (**E**) (t = 0.12, *p* = 0.91, unpaired *t*-test) and V1 activation (**F**) (F(1, 20) = 0.23, *p* > 0.05, two-way ANOVA with Tukey’s post hoc test) are illustrated as (**B**,**C**). ODI: ocular dominance index; MD: monocular deprivation; ipsi: ipsilateral eye; cont: contralateral eye. Data shown as mean ± SEM. n.s., not statistically significant.

To investigate the potential role of other interneurons, we applied AAV–LSL–shCdk5 in V1 of adult SST- or VIP-Cre mice (Figure 4A,D) and genetically reduced Cdk5 in SST or VIP interneurons (SST: shCdk5 = 0.31 ± 0.03, *p* < 0.0001; VIP: shCdk5 = 0.38 ± 0.03, *p* < 0.0001; unpaired *t*-test. Figure S2). However, four days of MD failed to induce a significant OD shift in SST-Cre (scramble = 0.16 ± 0.01, n = 5; shCdk5 = 0.15 ± 0.02, n = 6; unpaired *t*-test, *p* = 0.89; Figure 4B) or VIP-Cre mice (scramble = 0.16 ± 0.01, n = 6; shCdk5 = 0.16 ± 0.03, n = 6; unpaired *t*-test, *p* = 0.91; Figure 4E). Accordingly, the response magnitudes of the deprived or non-deprived eyes were also similar between mice with and without Cdk5 knockdown (SST: cont: scramble = 3.46 ± 0.10; shCdk5 = 3.57 ± 0.20; ipsi: scramble = 2.48 ± 0.05; shCdk5 = 2.58 ± 0.10; two-way ANOVA, *p* > 0.05; VIP: cont: scramble = 3.23 ± 0.23; shCdk5 = 3.13 ± 0.19; ipsi: scramble = 2.33 ± 0.18; shCdk5 = 2.25 ± 0.13; two-way ANOVA, *p* > 0.05; Figure 4C,F). In summary, these results suggest that adult mice can restore OD plasticity only by limiting Cdk5 expression in PV, but not SST, VIP or pyramidal, neurons in the visual cortex.

### 2.4. Cdk5 Blockade Reduces the Intrinsic Excitability of PV Neurons in the Visual Cortex

**Figure 5:**
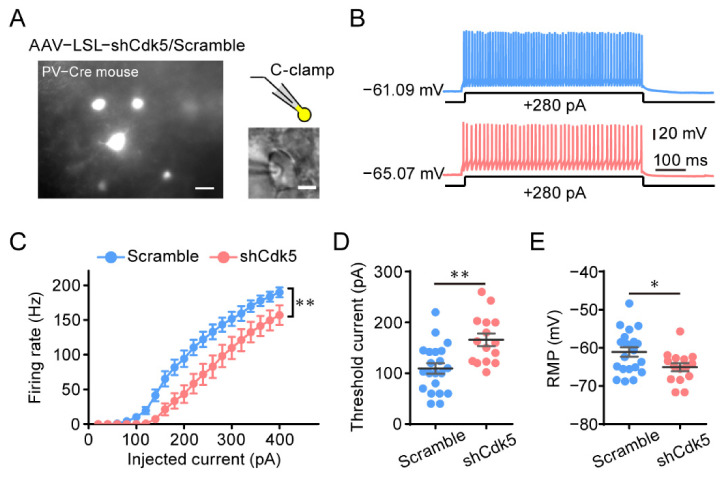
Decreased intrinsic excitability in PV neurons of the visual cortex after Cdk5 blockade. (**A**) Left: Photomicrograph of V1 area with virus-expressing PV neurons. Scale bar, 20 μm. Right, PV neurons for the whole-cell recording. Scale bar, 5 μm. (**B**) Representative trace in the whole-cell current clamp recording in PV neurons injected with AAV–scramble (blue) or AAV–shCdk5 (erythrinus) in response to 500 ms depolarizing current injection at 280 pA (scramble = 143.2 ± 10.12, n = 21; shCdk5 = 98.00 ± 16.04, n = 15; t = 2.50, *p* = 0.017, unpaired *t*-test). (**C**) The mean number of action potentials (AP No.) plotted against the eliciting currents (from 20 to 400 pA, +20 pA increment, over 500 ms) (F(1, 34) = 8.24, *p* = 0.007, two-way ANOVA). (**D**,**E**) The mean value of threshold current for AP generation (**D**) and resting membrane potential (**E**) in AAV–scramble- and AAV–shCdk5-injected PV neurons (threshold current: t = 3.53, *p* = 0.0012, unpaired *t*-test) (RMP: t = 2.37, *p* = 0.02, unpaired *t*-test). Data shown as mean ± SEM. * *p* < 0.05, ** *p* < 0.01.

To investigate the mechanism underlying the involvement of PV neurons in the reactivation of adult plasticity by Cdk5 inhibition, we performed patch clamp recordings of PV neurons four weeks after AAV–shCdk5 or AAV–scramble was injected into the visual cortex of adult PV-Cre mice (Figure 5A). In response to stepped somatic current injections, PV neurons showed a reduced firing rate of action potentials (APs) at each current intensity above rheobase in AAV–shCdk5 slices compared with control scramble (Figure 5B,C). At +100 pA current injection, the difference in firing rates began to reach a statistically significant level (scramble = 10.2 ± 3.6, n = 21; shCdk5 = 0.0 ± 0.0, n = 15; unpaired *t*-test, *p* < 0.05; Figure 5C). The decreased firing rate was accompanied by an increment in the threshold current for the generation of a single AP (rheobase) (scramble = 109.5 ± 10.3, n = 21; shCdk5 = 165.9 ± 12.3, n = 15; unpaired *t*-test, *p* < 0.01; Figure 5D) and a hyperpolarization of the resting membrane potential (RMP) (scramble = −61.1 ± 1.2, n = 21; shCdk5 = −65.1 ± 1.1, n = 15; unpaired *t*-test, *p* < 0.05; Figure 5E). These data indicate that specific downregulation of Cdk5 in PV neurons results in a decrease in the intrinsic excitability of PV neurons in the visual cortex.

### 2.5. Activation of Visual Cortex PV Neurons Attenuates the Restoration of OD Plasticity Induced by Cdk5 Inhibition

**Figure 6:**
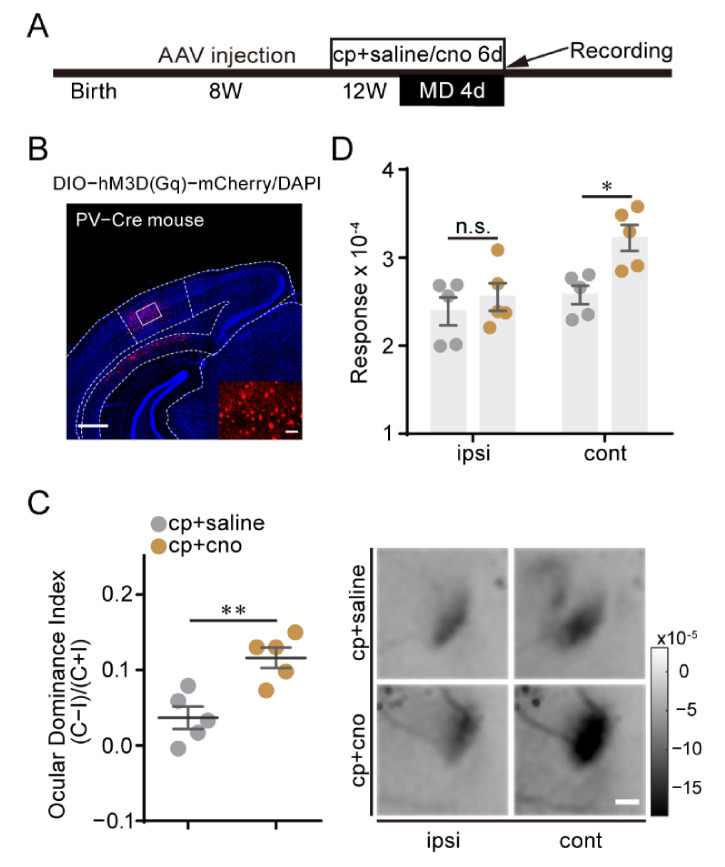
Activation of visual cortex PV neurons attenuates the restoration of OD plasticity induced by Cdk5 inhibition. (**A**) Experimental design. (**B**) A representative image of PV neurons infected with AAV–DIO–hM3Dq. Scale bar, 500 μm. Enlarged view shown in inset. Scale bar, 50 μm. (**C**) Left: comparison of ODI measured from PV-Cre mice treated with CP681301 + saline (gray) or CP681301 + CNO (brown) after 4d MD (t = 3.95, *p* = 0.004, unpaired *t*-test). Right: Example intrinsic signal images from CP681301 + saline- and CP681301 + CNO-treated PV-Cre mice after MD. Scale bar, 500 μm. (**D**) After 4d MD, comparison of response magnitude elicited by stimulating the ipsilateral eye or contralateral eye for CP681301 + saline- or CP681301 + CNO-treated PV-Cre mice (F(1, 16) = 7.87, *p* < 0.05, two-way ANOVA with Tukey’s post hoc test). ODI: ocular dominance index; MD: monocular deprivation; ipsi: ipsilateral eye; cont: contralateral eye. Data shown as mean ± SEM. n.s., not statistically significant, * *p* < 0.05, ** *p* < 0.01.

To validate the idea that the weakened activity of PV neurons by Cdk5 downregulation is necessary for the restoration of adult visual plasticity, we used the Designer Receptors Exclusively Activated by Designer Drugs (DREADD)-based chemogenetic tools to selectively express the engineered excited Gq-coupled receptor hM3Dq in PV neurons of the visual cortex. AAV–DIO–hM3Dq was injected into the visual cortex of adult PV-Cre mice. Four weeks later, clozapine N-oxide (CNO) or saline together with CP681301 solution was injected intraperitoneally for 6 days, starting 2 days before suturing the contralateral eye (Figure 6A,B). After monocular deprivation, the ODI of CNO-treated mice was significantly higher than that of control mice (CP + saline = 0.04 ± 0.01, n = 5; CP + CNO = 0.12 ± 0.01, n = 5; unpaired *t*-test, *p* < 0.01; Figure 6C). Measurements of response magnitudes showed that CNO treatment prevented loss of responsiveness to the deprived eye, while the non-deprived eye responses were comparable (cont: CP + saline = 2.58 ± 0.11; CP + CNO = 3.22 ± 0.15; ipsi: CP + saline = 2.39 ± 0.16; CP + CNO = 2.55 ± 0.16; two-way ANOVA, *p* < 0.05. Figure 6D). These results indicate that adult OD plasticity induced by Cdk5 downregulation can be attenuated by directly activating PV neurons of the visual cortex. Taken together, these findings suggest that the PV-based inhibitory circuit is the main target for Cdk5 to regulate visual plasticity in adult mice.

## 3. Discussion

In the visual cortex, PV interneurons are the largest subgroup of GABAergic neurons and target the perisomatic region of pyramidal neurons [32]. The maturity and activity of PV neurons are highly correlated with the development of the visual cortex [33,34,35]. Differently, SST interneurons target the distal dendrites of pyramidal neurons, and activation of SST interneurons can enhance OD plasticity in the adult mouse visual cortex [36]. VIP interneurons mainly innervate SST and PV interneurons and enhance the transmission of pyramidal neurons through disinhibition [37]. Long-term or short-term voluntary running enabled adult mice to maintain or restore juvenile-like plasticity, which may be related to the new inhibitory circuit remodeled by enhanced VIP activity [38,39]. In this study, we demonstrated that the cell-type specific knockdown of Cdk5 in PV neurons of V1, but not in SST or VIP neurons, can reactivate experience-dependent OD plasticity. Our findings support previous studies in that the same protein in different neuron types can play different roles in neural plasticity [40,41]. Specific deletion of methyl–CpG-binding protein 2 (MeCP2) in PV cells causes the absence of critical period plasticity, while the same operation on SST and pyramidal neurons is ineffective [40]. In another study, spine density and excitatory synapse number were reduced if ErbB4 was selectively ablated in parvalbumin-positive GABAergic interneurons, while there was no significant change by deletion or overexpression of ErbB4 in pyramidal neurons [41]. In general, we revealed that the restoration of OD plasticity by Cdk5 inhibition is mainly mediated through PV-based inhibitory circuits.

The relationship between Cdk5 activity and inhibition is still elusive. We showed that the specific downregulation of Cdk5 in PV neurons resulted in a decrease in the intrinsic excitability of PV neurons, which contributed to the restoration of OD plasticity in adult mice. Furthermore, short-term pharmacological inhibition of Cdk5 reduces the expression levels of glutamic acid decarboxylase and the frequency of mIPSC in pyramidal neurons [9]. Thus, our findings indicate a reduction in GABAergic inhibition after Cdk5 downregulation in the visual cortex, which is further supported by the result that DREADD activation of PV interneurons attenuates OD plasticity induced by Cdk5 inhibition. This supports the hypothesis that reduced GABAergic inhibition is associated with the restoration of OD plasticity in the adult visual cortex [17,18,21,22,23,24]. However, our result seems to contradict a previous study which found that genetic ablation of Cdk5 in *PVCre;fCdk5* mice leads to increased GABAergic transmission in CA1 [11]. This discrepancy may be due to differences in brain regions. Furthermore, long-term Cdk5 knockdown during brain development may cause the homeostatic regulation of GABAergic inhibition which is different from that caused by short-term knockdown in adulthood in our study. Therefore, it is worthwhile to examine the GABA activity of PV neurons in the visual cortex after knockdown of Cdk5 in future study.

The effects of the cell-specific Cdk5 knockdown reveal a previously unknown role of Cdk5 in regulating PV excitability. The activity of PV or Cdk5 is deregulated in various brain disorders including AD and schizophrenia, while little is known about their relation. Hyperactivity of Cdk5 is reported to contribute to the processing of AD [42]. Meanwhile, hyperexcitability of hippocampal PV interneurons is tightly correlated with memory impairment, and early intervention aimed at restoring PV interneuron activity rescues memory deficits and reduces amyloid plaque deposition in APP/PS1 mice [43]. In schizophrenic patients, the expression of the Cdk5 activator P35 is reduced in the prefrontal cortex and hippocampus [44]. In an adult schizophrenic mouse model, PV neurons are hypo-recruited, which is associated with cognitive deficits, which can be permanently rescued by chemogenetic activation of PV neurons, specifically in the ventral hippocampus or medial-prefrontal cortex [45]. Therefore, whether the modulation of Cdk5 on PV excitability is involved in the pathogenesis of these brain disorders is worthy to be investigated. The mechanisms underlying Cdk5 knockdown and the reduced excitability in PV interneurons are unknown. One possibility is that Cdk5 can directly modulate the activity of many ion channels by phosphorylation including potassium and calcium channels [46,47], which may be crucial for the excitability of PV neurons. Another possibility is that Cdk5 regulates the expression of certain proteins. For example, Cdk5 inhibition reduces OTX2 expression [9] as well as disrupting brain-derived neurotrophic factor activity-dependent gene transcription [48]. These proteins have been shown to be closely related to the maturation of PV neurons [49,50].

In summary, our findings suggest that the PV-based inhibitory circuit is the main target for Cdk5 in regulating the visual plasticity of adult mice. PV interneurons are crucial for maintaining a proper excitatory/inhibitory balance in the CNS and their deficits are associated with several psychiatric disorders. Thus, our finding that Cdk5 acts as a critical regulator of the intrinsic excitability of PV neurons may suggest potential treatment strategies for disorders associated with disturbance of PV neuron activity.

## 4. Materials and Methods

### 4.1. Animals

Animals were obtained from the Model Animal Research Center of Nanjing University or The Jackson Laboratory and bred at the animal facility of the University of Science and Technology of China (USTC). Adult male mice (8–13 weeks old) were used in all experiments. All animals were reared in standard cages, food and water were provided ad libitum and the animals were maintained on a 12 h:12 h light/dark cycle. All animal procedures were approved by the Institutional Animal Care and Use Committees at USTC and the Chinese Academy of Sciences.

### 4.2. Drug Administration

C57BL/6J adult mice were injected, twice a day, with CP681301 (5.8 mg/kg, i.p.; kindly provided by Pfizer) or vehicle (1% DMSO, 1% Tween-80, in normal saline). Clozapine N-oxide (CNO) was systematically co-injected with CP681301 at a dose of 5 mg/kg/day for 6 days to activate hM3Dq virus in PV neurons.

### 4.3. Virus Injection

Cre-dependent adeno-associated virus (AAV) was used in this study. The sequence used for RNAi targeting Cdk5 was CGGGAGATCTGTCTACTCAAA. The shCdk5 sequence was converted into a miR30 and cloned into a vector containing a CMV promoter-driven eGFP. A sequence of loxp-Stop-loxp (LSL) was designed in front of the shCdk5 sequence, and the shCdk5 sequence was expressed after the LSL sequence was cut off by Cre recombinase. AAV that expressed shRNA targeting a non-specific sequence (CGCTGAGTACTTCGAAATGTC) was used as a control (scramble). AAV–DIO–hM3Dq–mCherry and AAV–DIO–mCherry were used to manipulate the activity of PV neurons. All viruses were purchased from Obio Technology company of Shanghai.

Mice were initially anesthetized with 4% isoflurane in oxygen, and 2% for maintenance. The scalp of mice was incised along the midline, and a small burr hole was created with a dental drill at the following stereotaxic coordinates: 1.0 mm anterior to lambda, 2.75 mm lateral from midline. A volume of 400 nL of virus was injected 0.5–0.6 mm below the cortical surface at 20 nL/min at each site through a 1 μL microsampler. After finishing each injection, the pipette was left in position for 5 min to prevent leakage. To assess the efficacy of RNAi, one side of V1 was injected with AAV–shCdk5 or AAV–DIO–hM3Dq, and the other side was injected with AAV–scramble or AAV–DIO–mCherry. After removing the pipette from the injected point, the scalp was sutured with a sterile suture. Animals were allowed to recover from anesthesia and were returned to their home cages once they showed regular locomotion.

### 4.4. Monocular Deprivation

Monocular deprivation (MD) was performed by suturing the eyelid of adult mice under 1–3% isoflurane anesthesia. The eye lid contralateral to the imaged V1 was sutured shut with 6–0 nylon sutures. Mice were returned to their former home cages and checked daily until the recording day. Mice whose sutured eye reopened or those with any indications of cataract were removed from the study.

### 4.5. Optical Imaging of Intrinsic Signals

Surgical preparation, visual stimuli, optical imaging of intrinsic signals and data analysis were carried out as described previously [16,30]. Mice were anesthetized with isoflurane in an airtight jar supplemented with an intramuscular injection of chlorprothixene (10 mg/kg i.m.). Then, the mice were quickly head fixed using a stereotaxic apparatus when they were anesthetized. Isoflurane was maintained with a constant level of (0.75%) in O_2_ (flow rate: 1.0 L/min) when the mice were stable. The temperature was maintained at 37 °C with a heating pad. A small incision of the skin was created to expose the area of the visual cortex. The skull of the recording area was ground thin, cleaned and covered with silicone oil. The recorded area was illuminated with green light of 550 nm to obtain a brain vasculature map, and red light of 630 nm to acquire evoked responses. Images were captured using a Dalsa Pantera 1M60 CCD camera. Visual stimuli were created by MATLAB (Mathworks, Inc., Natick, MA, USA) using the Psychophysics Toolbox [51,52]. A visual stimulus consisting of a horizontal bar moving in a vertical direction (upward or downward) was displayed in the binocular visual field (−5°–15° azimuth) to elicit responses in the binocular zone of V1. For each eye, cortical activity elicited at the stimulus frequency was calculated by Fourier analysis. The magnitudes of response are fractional changes in reflectance. In each session, a set of four images was taken by visualizing the response of each eye (i.e., the ipsilateral or contralateral eye) to each direction (i.e., upward or downward). Maximum magnitude values are presented as a measure of response strength separately for each eye. Ocular dominance index was calculated as an average of (Contra − Ipsi)/(Contra + Ipsi) from each pixel in a region of interest, which was manually selected on the basis of raw signals from ipsilateral eye responses with a threshold at 40% of the peak magnitude.

### 4.6. Brain Slice Preparation and Recording

Brain slices were prepared from adult PV-Cre mice after AAV was expressed for 4 weeks. Mice were sacrificed by decapitation after being deeply anesthetized with pentobarbital sodium. The whole brain was immediately removed from the skull. Coronal slices (300 μm thick) containing V1 were prepared using a vibrating microslicer (VT1200s, Leica, Microsystem, Wetzlar, Germany) in ice-cold N-methyl-D-glucamine artificial cerebrospinal fluid (NMDG ACSF) containing the following (in mM): 93 N-methyl-D-glucamine (NMDG), 2.5 KCl, 1.2 NaH_2_PO_4_, 30 NaHCO_3_, 20 HEPES, 25 glucose, 2 thiourea, 5 Na-ascorbate, 3 Na-pyruvate, 0.5 CaCl_2_, 10 MgSO_4_ and 3 glutathione (GSH) (osmolarity: 300–305 mOsm/kg), bubbled with 95% O_2_/5% CO_2_. Slices were initially incubated in NMDG ACSF for 11 min at 32 °C and then were immediately transferred to N-2-hydroxyethylpiperaxine-N-2-ethanesulfonic acid (HEPES) ACSF at 30 °C for 1 h before recordings. The HEPES ACSF contained (in mM): 92 NaCl, 2.5 KCl, 1.2 NaH_2_PO_4_, 30 NaHCO_3_, 20 HEPES, 25 glucose, 2 thiourea, 5 Na-ascorbate, 3 Na-pyruvate, 2 CaCl_2_, 2 MgSO_4_ and 3 glutathione (GSH) (pH: 7.4, osmolarity: 300–305 mOsm/kg). All recordings were performed at 30 °C. PV neurons expressing AAV-shCdk5 or AAV-scramble from the visual cortex were visualized using an upright microscope (FN1, Nikon, Tokyo, Japan) equipped with a fluorescence camera. Whole cell current clamp techniques were used to record the PV neurons. The pipette solution contained (in mM): 130 K-gluconate, 2 MgCl_2_, 5 KCl, 0.6 EGTA, 10 HEPES, 2 Mg-ATP and 0.3 Na-GTP with osmolarity adjusted to 285–290 mOsm/kg and pH adjusted to 7.2 with KOH. The bath solution (ACSF) contained the following (in mM): 129 NaCl, 3 KCl, 2.4 CaCl_2_, 3 HEPES, 1.3 MgSO_4_, 1.2 KH_2_PO_4_, 20 NaHCO_3_ and 10 glucose (pH: 7.4, osmolarity: 300–305 mOsm/kg), when saturated with carbogen (95% O_2_ and 5% CO_2_). The perfusion rate of the bathing solution was 2.5 mL/min.

Electrophysiological recordings were carried out in somata with a HEKA EPC9 amplifier (HEKA Electronics, Lambrecht/Pfalz, Germany). The following parameters were measured and analyzed: (1) the resting membrane potential, (2) AP threshold current (current threshold for single AP generation, 500 ms duration), (3) AP firing rates. We excluded data for analysis when the series resistance exceeded 20 MΩ.

### 4.7. Immunohistochemistry

Mice were anesthetized by injecting a mixture (i.p.) of ketamine (0.1 mg/g) and xylazine (0.01 mg/g) and transcardially perfused with ice-cold 0.01 M PBS, followed by 4% paraformaldehyde dissolved in 0.01 M PBS. Brains were extracted and post-fixed in 4% paraformaldehyde overnight at 4 °C and then cryoprotected with 30% sucrose in 0.1 M PBS for 48 h. Coronal sections were cut at 40 μm thickness on a cryostat microtome (CM1950, Leica, Microsystem, Wetzlar, Germany). Free floating sections were permeabilized and blocked with 0.5% Triton X-100 and 5% BSA for 1.5 h at room temperature. After that, sections were immunostained by incubation with rabbit anti-Cdk5 (1:100, Abcam, ab40773) and anti-guinea pig parvalbumin (1:500, SYSY, 195 004) for 24–48 h at 4 °C. After washing with 0.01 M PBS, sections were conjugated with a suitable Alexa 594 secondary antibody (goat anti-rabbit, 1:500, Jacksonimmuno) and PV (donkey anti-guinea pig-Cy3, 1:500, Jacksonimmuno) for 2 h at room temperature. After counterstaining with the nuclear dye Hoechst 33342 (2 μg/mL, Sigma, B2261) for 10 min to distinguish each sublayer, the brain slices were transferred to a glass slide and mounted with an anti-quenching agent (Vector CA 94010). Photographing was performed under constant parameters of the Zeiss LSM 880 confocal laser scanning microscope. In order to test the effect of Cdk5 inhibition on protein expression, we selected the slices which expressed AAV–shCdk5 in one side of the visual cortex, and AAV–scramble in the other side.

### 4.8. Statistical Analysis

All of the data were evaluated using GraphPad Prism 6.0 (GraphPad Software, California, USA). Differences between two groups were analyzed using a two-tailed unpaired *t*-test. To determine differences among multiple groups, two-way ANOVA followed by Tukey’s test was performed. All statistical details are described in the figure legends. Data are represented as mean ± SEM. *p* < 0.05 was considered statistically significant.

## Data Availability

The data analyzed and presented in this study are available from the corresponding author on request.

## Supplementary Materials

The following are available online at https://www.mdpi.com/article/10.3390/ijms23010186/s1.
