# Supplementary material for: Inhibition of Cdk5 in PV Neurons Reactivates Experience-Dependent Plasticity in Adult Visual Cortex
Source: Int J Mol Sci. 2021 Dec 24;23(1):186. doi: 10.3390/ijms23010186 (PMC8745415; doi:10.3390/ijms23010186)
Supplement: Supplementary file 1 [file ijms-23-00186-s001.zip › ijms-1485998-supplementary.pdf]

International Journal of *Molecular Sciences*

MDPI

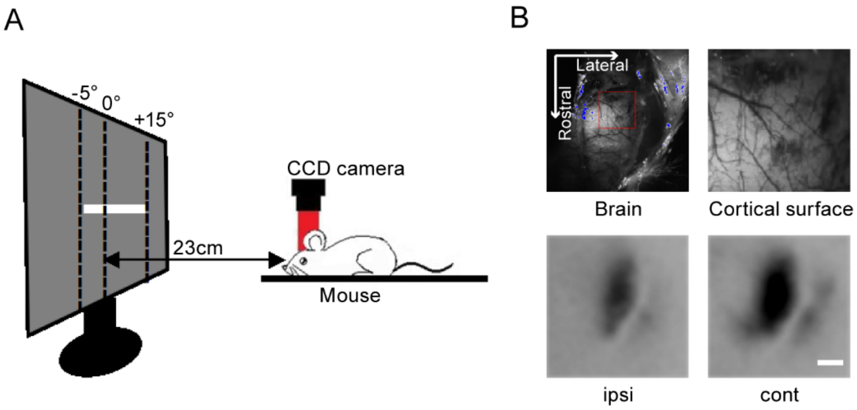


**Figure S1.** Experimental technique used for OD plasticity measurement. (A) Left: Schematic for imaging of intrinsic signals. (B) Top: Cortical blood vessel pattern was captured under green light. Bottom: Example response magnitude for stimulation in the binocular visual field under red light. Scale bar, 500 μm.

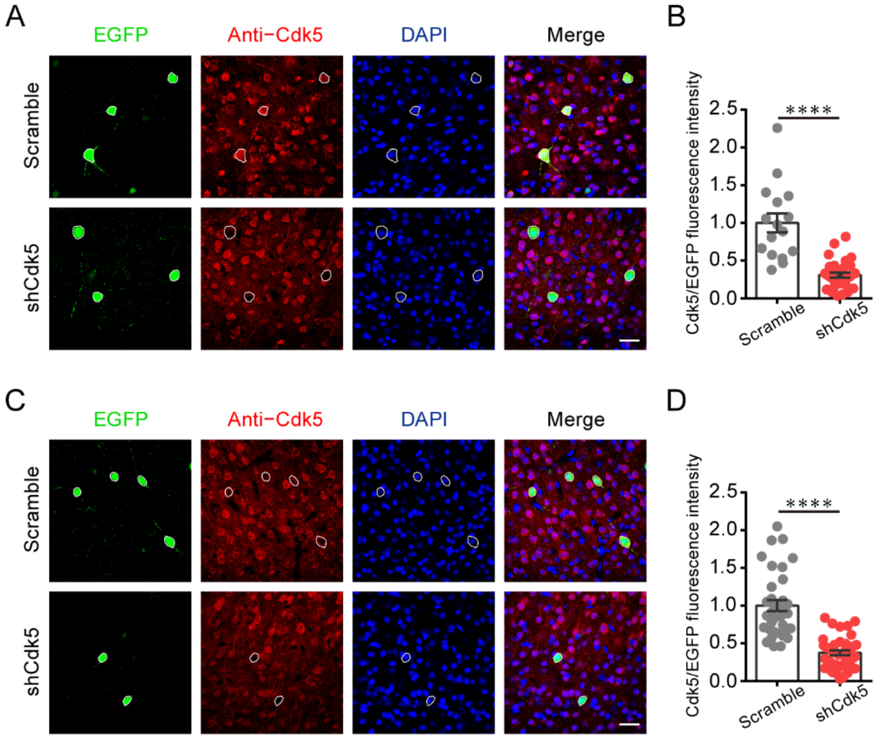


**Figure S2.** Genetic reduction in Cdk5 in SST or VIP neurons of the visual cortex. (A, C) IHC in V1 of SST- and VIP-Cre mice, virus-infected SST and VIP neurons (green) and Cdk5 immunoreactivity (red). Virus-infected neurons are enclosed by white hollow circles. Scale bar, 30 μm. (B, D) Cdk5 was quantified by immunofluorescence using Abs against Cdk5 (SST: scramble = 1.00 ± 0.13, n = 16; shCdk5 = 0.31 ± 0.03, n = 31; t = 6.86, $p < 0.0001$, unpaired *t*-test; VIP: scramble = 1.00 ± 0.07, n = 35; shCdk5 = 0.38 ± 0.03, n = 42; t = 8.32, $p < 0.0001$, unpaired *t*-test). Data shown as mean ± SEM.
